# An Assessment of the Social Determinants of Health in an Urban Emergency Department

**DOI:** 10.5811/westjem.2021.4.50476

**Published:** 2021-07-15

**Authors:** Edgardo Ordonez, Katherine Dowdell, Natasha M. Navejar, Deepa Dongarwar, Aya Itani, Hamisu M. Salihu

**Affiliations:** * Baylor College of Medicine, Henry J.N. Taub Department of Emergency Medicine, Houston, Texas; † Baylor College of Medicine Center of Excellence in Health Equity, Training and Research, Houston, Texas

## Abstract

**Introduction:**

Social determinants of health (SDOH) have significant impacts on patients who seek care in the emergency department (ED). We administered a social needs screening tool and needs assessment survey to assess SDOH and evaluate for trends in the population of patients visiting our ED.

**Methods:**

A survey was distributed via convenience sampling to adult ED patients to capture self-reported demographic information and data about social needs. We categorized the questions related to SDOH based on the International Classification of Diseases, Tenth Revision, Clinical Modification coding format and created a composite variable called “SDOH Strata” based on the SDOH Index scores (0–5-low, 6–10-middle, or ≥11-high). We conducted bivariate analyses using the sociodemographic characteristics of the patients and their SDOH Strata using Fisher’s exact test. We then conducted multinomial logistic regression to examine the association between the patients’ sociodemographic characteristics and the SDOH Strata.

**Results:**

A total of 269 surveys were collected. We observed that Hispanic/Latino patients were more than two times as likely (odds ratio: 2.04, 95% confidence interval [CI], 1.12,−6.51) to be in the higher impact stratum than in the lower impact stratum. Those who were undocumented had 3.43 times increased adjusted odds (95% CI, 1.98, 9.53) of being in the higher than the lower impact stratum compared to US citizens. Additionally, people speaking Spanish as their primary language were 5.16 times as likely to be in the higher impact stratum compared to the reference (English-speaking and lower impact stratum).

**Conclusion:**

In our patient population, patients noted to have the highest impact burden of the SDOH were those who identified as Hispanic/Latino, Spanish-speaking, and undocumented immigrant status.

## INTRODUCTION

The Emergency Medical Treatment and Labor Act (EMTALA) enacted by the US Congress in 1986 mandates that anyone coming to an emergency department (ED) has the right to be stabilized and treated, regardless of ability to pay or insurance status.^1^ Many EDs serve as a safety net for those who have unmet social needs and these EDs are often located where vulnerable patient populations seek care, including those who are uninsured and undomiciled. As the gateway to the healthcare system, the ED is in a prime position to assess patients’ social needs and help formulate plans to address them. Previously, ED interventions aimed at addressing patients’ social needs such as healthcare access, insurance enrollment, and patient follow-up adherence have been found to be successful. Interventions have included the use of social workers, community health workers, and student volunteers to provide linkages to local resources.^2^–^6^ While it may seem counterproductive to address non-emergent issues in the ED setting, the EDs relevance in addressing these issues is clear. Thus, a new area of focus, “social emergency medicine,” has been established to incorporate social context into the structure and practice of emergency care.^7^,^8^

Social determinants of health (SDOH) are defined as “the conditions in which people are born, live, learn, work, play, and age.”^9^ They are divided into five determinant areas: 1) economic stability; 2) education; 3) social and community context; 4) health and healthcare; and 5) neighborhood and built environment.^10^ Unmet social needs, such as food, housing, transportation, and other societal factors including substance use disorder, domestic violence, mental illness, and limited English proficiency, are known to have a significant impact on healthcare outcomes. Understanding an individual’s disease or diagnosis alone may not be sufficient to positively impact their health. For clinicians and interdisciplinary healthcare teams, addressing social needs is necessary to have a positive effect on health and help eliminate health inequities.

The SDOH have significant impacts on patients who seek care in the ED. Economic stability affects employment, housing status, and food security, and can have significant downstream effects on overall health. Studies have shown that there is a higher prevalence of poor health and mortality in the unemployed.^11^–^13^ In homeless individuals, lack of resources makes it difficult to maintain health and navigate the health system, and makes them more likely to use the ED than the general population.^14^–^17^ Food insecurity, lower education levels, and limited access to primary care have all been found to increase ED utilization as well.^18^–^23^ In one study, Spanish-speaking patients with limited English proficiency (LEP) were found to have increased unplanned ED revisits within 72 hours.^24^ Poor health literacy has also been associated with medication nonadherence, overall poorer health, increased ED utilization, and increased hospitalization.^25^–^27^

When negatively impactful SDOH are prevalent, they present challenges to the health of a significant portion of the population. Patients are often left to seek solutions in the ED setting. Many EDs that function as safety-net hospitals regularly care for the underserved and vulnerable populations. These patients may comprise the entire spectrum of the socioeconomically disadvantaged, which may include the homeless, the uninsured, and the unemployed. We administered a social needs screening tool and needs assessment survey to evaluate trends in our patient population to gain a broader understanding of the community needs and impacts of the SDOH.

## METHODS AND ANALYSIS

We developed a survey to capture self-reported sociodemographic data along with information on SDOH in ED patients. Sociodemographic questions included age, gender, race/ethnicity, citizenship status, and sexual identity, among others. We incorporated questions from the previously validated Centers for Medicare & Medicaid Services’ Accountable Health Communities Health-Related Social Needs Screening Tool to obtain information on numerous SDOH such as housing, social support, and substance use.^28^ We added questions regarding ED utilization, including the participants’ reasoning for selecting the ED for care and barriers to accessing healthcare. The survey tool was piloted on a sample of 15 patients by our research staff to ensure the questions were easy to understand. Minor suggestions on wording for two questions were made and they were revised. The pilot data was not included as part of the survey analysis.

**Population Health Research Capsule**

What do we already know about this issue?

*Social determinants of health (SDOH) have significant impacts on patients seeking care in the ED. Social needs screening can help formulate targeted interventions*.

What was the research question?

*We sought to assess the SDOH in ED patients in a safety-net hospital and identify patients with the highest impact burden of SDOH*.

What was the major finding of the study?

*The highest burden of SDOH was in patients who identified as Hispanic/Latino, Spanish-speaking, and undocumented immigrants*.

How does this improve population health?

*Our study points to the need to assess the SDOH in ED patients using multidisciplinary teams to identify social needs and help design strategies to address them*.

Patients recruited for the survey were registered in the ED of a large, urban safety-net hospital located in Houston, Texas. Participants were recruited voluntarily using a convenience sample in the ED, including the waiting room and various lower acuity care areas shortly after a medical screening examination or being bedded to a room, during February to early March 2020. Recruitment was done between the hours of 9 am and 11 pm Monday through Sunday, depending on the availability of research staff. Excluded patients were those under the age of 18, with 1:1 sitters, and incarcerated individuals. Pregnant patients > 20 weeks were also not included as they go directly to the obstetrics intake unit. Surveys were administered verbally by trained research staff in a private screening room in the waiting area or individual patient rooms in a care area. Individual responses were entered into a secure database via smartphone or tablet. The survey was translated into Spanish, and phone interpreters were available for patients who did not speak English.

We performed descriptive statistics on the sociodemographic information of the survey respondents, and present the results with frequency tabulations and percentages. We categorized the questions related to SDOH based on the *International Classification of Diseases, Tenth Revision, Clinical Modification* coding format, whenever possible, or grouped them into more meaningful categories. We also performed descriptive statistics on the questions related to the SDOH. Next, we dichotomized the responses to all SDOH questions into the following groups: 1) 0 – does not contribute to poor SDOH; and 2) 1 – contributes to poor SDOH. We created a SDOH index by summing the scores of all the SDOH questions for each of the respondents. The lower the SDOH index score, the lesser the individual was impacted by poor SDOH. We created a composite variable called “SDOH Strata” based on the SDOH index scores. A modified Delphi process was used with experts in emergency medicine, health disparities, and epidemiology to discuss how to stratify the SDOH index score to create the strata. We categorized the SDOH index scores as follows: 0–5 “lower impact stratum;” 6–10 “moderate impact stratum;” and scores ≥11 were categorized into the “higher impact stratum.” For example, if a person belonged to the higher impact stratum, they would be considered to have a higher SDOH burden as compared to those in the moderate or lower impact strata.

We conducted bivariate analyses using the sociodemographic characteristics of the patients and their SDOH strata using Fisher’s exact test, as some of the frequency values were very small (ie, less than five). Due to the small percentage of missing information (only six records), no imputation techniques were applied, and we removed the missing records prior to running multivariate analyses. Lastly, we conducted multinomial logistic regression to examine the association between the patients’ sociodemographic characteristics and the SDOH strata. The lower stratum was considered the referent category. All tests of hypothesis were two-tailed with the type-1 error rate set at 5%. The institutional review board deemed this survey a quality assurance activity as the project’s focus was to identify the social needs of patients using our ED for program planning and implementation, and no identifying information was collected.

## RESULTS

A total of 269 patients agreed to participate in the survey, and 263 completed it in its entirety. Patients who declined participation were excluded from the analysis. For reference, our total ED volume for 2019 was just under 82,000 visits. Table 1 summarizes the self-reported sociodemographic characteristics of the 269 patients. The age distribution was generally young, with 86.2% of our sample under the age of 60. In 2019, 43.3% of our ED population was between 18–39, which is comparable to our study population of 43.9%. Additionally, 39.8% of our ED population was between 40–59, which is comparable to our study population of 40.1%. Hispanic/Latinos (46.1%) and Blacks (34.9%) comprised our representative sample. For comparison, our overall ED population for 2019 was 56% Hispanic/Latino and 33% Black. The numbers of English and Spanish speakers were nearly equivalent at 128 and 122, respectively.

The supplemental table shows the relative distribution of SDOH affecting this population, which we classified as problems related to education and literacy, housing and economic circumstances, psychological trauma, employment, social environment, substance abuse, mental health, or access to healthcare. The majority of our patients (43.9%) had earned a high school diploma or equivalent, whereas 22.3% had less than a high school-level education. While 75.1% reported having a stable place to live, 74.7% also reported living in poor conditions. In terms of access to food, 51.7% of respondents reported food insecurity or shortage. Financial insecurity was reported by 69.9% of participants. Regarding employment, 17.1% of those surveyed were unwillingly out of work, and another 13.8% were unable to work due to a disability, contributing to less favorable SDOH.

Substance abuse was not uncommon in our patient population, particularly the use of alcohol (five or more drinks in a day in males or four or more drinks in a day in females) and tobacco (any use). Rates of tobacco use and alcohol binge drinking were comparable, with 32.3% and 32.7% of patients admitting to each activity in the past year, respectively. Mental health challenges were also prevalent, as 46.5% of patients experienced feeling down, depressed, or hopeless at least several days in the prior two weeks. This population experienced significant barriers in accessing healthcare, with 67.7% experiencing a barrier of at least one kind, most commonly lack of insurance. There was also a significant proportion of patients who came to the ED for reasons that reflected poor SDOH: concern about the cost of other facilities (11.5%); lack of awareness of alternative options (4.1%); or “other” (ie, no access to reliable transportation to a more appropriate facility, unavailability of a timely outpatient appointment, new to the area, and lacking a primary care doctor) (24.9%). Table 2 displays the sociodemographic characteristics of patients in relation to their SDOH stratum, with lower impact stratum meaning more optimal SDOH and higher impact stratum meaning less optimal SDOH. The lower, middle, and higher impact strata represented 47 (17%), 118 (44%), and 104 (39%) patients, respectively. Thus, the majority of patients had to deal with multiple sub-optimal conditions that contributed to poor SDOH and, therefore, a higher impact score. Notably, the middle and higher strata contained a similar proportion of males and females, while the lower impact stratum had a predominance of females (63.8%). Race/ethnicity of patients also varied markedly between strata. Being White was disproportionately weighted toward lower and middle impact strata (81.3%). By contrast, Hispanic/Latinos and Blacks were more likely to fall within the higher impact strata (46.0% and 33%, respectively). This trend held for immigrants, especially those who were undocumented. US citizens, conversely, were more evenly dispersed across strata and comprised the bulk of the lower impact stratum (89.4%). The primary language spoken also appeared to be a predictor of stratum, with Spanish speakers expressing more SDOH burden than their English-speaking counterparts. The higher impact stratum was 56.7% Spanish speakers, which was in stark contrast to the lower impact stratum, where English speakers held a 74.5% majority.

Table 3 shows the results of multinomial logistic regression between the various sociodemographic characteristics of patients and their likelihood of being in the middle or higher impact stratum as compared to the referent category of those in the lower impact stratum. We observed that when compared to White patients, Black patients were 1.22 times as likely (odds ratio [OR]: 1.22, 95% confidence interval [CI], 1.08–1.76), and Hispanic/Latino patients were two times as likely (OR: 2.04, 95% CI, 1.12, 6.51) to be in the higher impact stratum. Those who were undocumented had 3.43 times increased adjusted OR (aOR) (95% CI, 1.98, 9.53) of being in the higher rather than the lower impact stratum compared to US citizens; whereas being lawfully present or a non-immigrant (student visa, temporary employee, visitor) had an 82% reduced aOR: 0.18 (95% CI, 0.05, 0.63) of being in the higher impact stratum compared to the referent groups. Additionally, people having Spanish as their primary language were 3.12 times as likely to be in the middle impact stratum but 5.16 times as likely to be in the higher impact stratum compared to the reference (English speaking and lower impact stratum).

## DISCUSSION

In this study population, the sociodemographic factors with the most significant association to a high burden from social needs included being Hispanic/Latino, primarily Spanish-speaking, and undocumented immigrant status. Numerous factors have been postulated as a link between undocumented status and increased social needs impact, including discrimination, immigration policy, and a lack of understanding of the US healthcare system by immigrant populations.^29^ A paper by Gurrola and Ayon in 2018 eloquently outlines the far-reaching consequences of anti-immigration policy and structural discrimination against undocumented immigrants regarding each of the five SDOH domains. A common theme affecting each domain was lack of integration, preventing equal educational opportunities, economic stability, and access to basic healthcare services.

Similarly, there have been several studies seeking to identify barriers faced by patients with a primary language other than English or LEP persons. A study by Sentell suggested that LEP individuals may be less likely to receive or be recommended for critical resources.^30^ This study focused on access to mental health services among Latinos and Asian/Pacific Islanders. Stark disparities existed when controlling for ethnicity in each group identifying LEP as the primary risk factor in lack of mental health referral.

Numerous studies have highlighted the risks of social and health inequity faced by minority populations in the US. However, as suggested by Lillie-Blanton and LaVeist, the relationship between minority status and socioeconomic status is so complex that controlling for social factors and attributing risk purely to race or ethnicity alone may completely miss the point.^31^ This survey has helped to identify vulnerable groups within our specific patient population and opens the door to future-focused projects. Moving forward requires an action plan such as that suggested by Wong et al: “[T]o design services that promote health equity, there must be a clear focus on specific communities at risk, a commitment to listen and collect meaningful data to understand local needs and priorities, a conviction to make progress, and ongoing assessment of health outcomes.”^32^

While screening is often the initial step in understanding the impact of SDOH within a population, what is known regarding the state of SDOH screening in the US? With widespread knowledge of the impact of SDOH and commitment to improving health outcomes, there has been an increase in screening programs that vary in terms of care setting, topics addressed, and linkage to resources.^33^ There is currently a lack of consensus guidelines on a particular screening tool with numerous in use.^34^ A portion of the screening tool used by this group was the Accountable Health Communities Health-Related Social Needs Screening Tool, as the tool has been tested in a multitude of communities to date.^28^ However, it must be noted that novel questions were added to this survey, given our desire for sociodemographic data in conjunction with the domains of social determinants.

In addition to choosing a survey tool, there was discussion regarding where screening could or should take place.^34^ The ED may be the only point of contact within a healthcare system for patients with the highest burden of social needs. To eliminate health disparities and achieve health equity, interdisciplinary teams that include physicians, nurses, social workers, counselors, community health workers, and volunteers, should collaborate and coordinate to address the patient’s social needs. Much of this work can be done through increased community engagement and advocacy for systemic and social change when there are unmet needs in vulnerable populations.

In an article by Hsieh, the argument is made that the focus in the ED must shift to include social needs in the acute care setting to truly optimize healthcare costs and health outcomes.^35^ However, a paradox exists in addressing social needs in a busy, fast-paced setting. We would like to propose a few thoughts on how SDOH screening may be implemented into the ED workflow. One initial consideration for implementation is how to create a program that will be attainable for departments with varying resources. For instance, a plan would ideally be actionable for any setting, from an academic center with 24-hour social work coverage to a community ED with minimal interdisciplinary support. As previously mentioned, it would be ideal to engage support staff and avoid developing a burdensome task for the busy emergency physician. Wallace et al published a proposed workflow for SDOH screening worth highlighting, as several crucial points were analyzed in that study.^36^ One initial question was who would be responsible for administering the screening questions. Numerous individuals were considered, including ED registration and nursing, with the ultimate decision to use registration staff. The screening tool used included 10 questions targeting SDOH domains known to be actionable by available resources. Once screened, the protocol outsourced referrals to existing state 2-1-1 systems. Our study provides a carefully thought-out workflow, but it was only active at a single institution. Necessary follow-up would entail a multicenter trial of a workflow tracking short- and long-term outcomes.

## LIMITATIONS

While this survey was intended to be a needs assessment, several inferences were made from the results. There are, however, flaws in making definitive conclusions from an analysis of this type of investigation. First, because it was a single-center study, it lacks external validity. There were also limitations in the method of gathering participants using a convenience sample. Convenience sampling can result in sampling bias, which would not necessarily be representative of the population being assessed and can similarly affect selection bias, which may not reflect true similarities or differences in respondent groups. Surveys were not completed 24 hours a day, which could have affected the sample of patients enrolled. As we intended to provide actionable program planning based on completed survey results, only patients who agreed to respond to the survey were included; thus, the total number of patients approached was not tracked. To be more representative of our population, we would have benefitted from a more systematic and random sampling methodology.

Our survey was administered at a safety-net hospital, which can overestimate individuals with socioeconomic constraints. Most patients who use our health system are referred to us because they are known to be uninsured or experiencing financial hardships. This survey was important in identifying the social needs of our specific patient population, but there can also be the issue of self-reporting. None of our responses were collected from the electronic health record, which may have caused two types of self-reporting bias: social desirability bias and recall bias. Questions were not asked about marital status, household size, and childcare issues. Also, there was limited analysis of socioeconomic constraints, as the association of household income to the SDOH was not evaluated. Due to ease of recruitment, languages spoken were primarily English and Spanish, which prevented a thorough assessment of the prevalence of specific SDOH in non-English speakers.

Lastly, the COVID-19 pandemic limited the number of surveys we were able to administer due to operational changes in our ED workflow. Our care areas were geographically adjusted, and patient access was limited to essential providers, which prevented us from deploying some of our research staff. The stay-at-home orders in early March 2020 required us to terminate survey collection. The pandemic would likely have had a significant shift in the results from surveys completed before the economic shutdown and social distancing directives.

## CONCLUSION

In our patient population, those who identified as Hispanic/Latino, Spanish-speaking, and undocumented immigrant status were noted to have the highest burden of social determinants of health. Our study was limited primarily by its small size at a single center and the lack of random sampling, which would have improved the generalizability of our findings. Even so, this points to the need to address the SDOH in patients who present to the ED for care, as for many, SDOH can prove burdensome and significantly affect health outcomes.

## Figures and Tables

**Table 1:** Sociodemographic characteristics of patients surveyed.

Sociodemographics	N	%
Age		
18–39 years	118	43.9%
40–59 years	108	40.1%
60–79 years	37	13.8%
80+ years	2	0.7%
Missing	4	1.5%
Gender		
Female	146	54.3%
Male	118	43.9%
Other/Missing	5	1.9%
Identify as LGBTQ+		
No	242	90.0%
Yes	15	5.6%
Other/prefer not to answer	12	4.5%
Race/Ethnicity		
White	32	11.9%
Black	94	34.9%
Hispanic	124	46.1%
Others	14	5.2%
Missing	5	1.9%
Citizenship status		
US citizen	167	62.1%
Lawfully present	36	13.4%
Undocumented	44	16.4%
Non-immigrant	7	2.6%
Prefer not to answer/missing	15	5.6%
Type of Insurance		
Medicare	16	5.9%
Medicaid	8	3.0%
CHIP	4	1.5%
Private	14	5.2%
Financial Assistance Program	107	39.8%
Others	11	4.1%
Uninsured	109	40.5%

*LGBTQ+*, lesbian, gay, bisexual, transgender, queer, and other; *US*, United States; *CHIP*, Children’s Health Insurance Program.

**Table 2:** Sociodemographic characteristics of patients visiting the emergency department based on their social determinants of health (SDOH) stratum (based on SDOH index score: lower impact stratum – index score 0–5; middle impact stratum – index score 6–10; and higher impact stratum – Index score 11–16).

	Lower impact stratum		Middle impact stratum		Higher impact stratum	
		
	N	Prevalence	N	Prevalence	N	Prevalence
Total	47		118		104	
Age						
18–39 years	17	14.4%	53	44.9%	48	40.7%
40–59 years	26	24.1%	45	41.7%	37	34.3%
60+ years	4	10.3%	19	48.7%	16	41.0%
Missing	0	0.0%	1	25.0%	3	75.0%
Gender						
Female	30	20.5%	65	44.5%	51	34.9%
Male	16	13.6%	52	44.1%	50	42.4%
Other/missing	1	20.0%	1	20.0%	3	60.0%
Identify as LGBTQ+						
No	39	16.1%	107	44.2%	96	39.7%
Yes	6	40.0%	5	33.3%	4	26.7%
Other/prefer not to answer	2	16.7%	6	50.0%	4	33.3%
Race/Ethnicity						
White	12	37.5%	14	43.8%	6	18.8%
Black	20	21.3%	43	45.7%	31	33.0%
Hispanic/Latino	14	11.3%	53	42.7%	57	46.0%
Others	1	7.1%	7	50.0%	6	42.9%
Missing	0	0.0%	1	20.0%	4	80.0%
Citizenship status						
US citizen	42	25.1%	71	42.5%	54	32.3%
Lawfully present/non-immigrant	3	7.0%	18	41.9%	22	51.2%
Undocumented	2	4.5%	22	50.0%	20	45.5%
Prefer not to answer/missing	0	0.0%	7	46.7%	8	53.3%
Primary language						
English	35	27.3%	56	43.8%	37	28.9%
Spanish	11	9.0%	52	42.6%	59	48.4%
Other	1	5.3%	10	52.6%	8	42.1%

*LGBTQ+*, lesbian, gay, bisexual, transgender, queer, and others; *US*, United States.

**Table 3:** Multinomial logistic regression between sociodemographic characteristics of patients visiting the emergency department and their social determinants of health stratum (lower impact stratum is the referent group).

Sociodemographics	Middle Impact Stratum [OR] (95% CI)	Higher Impact stratum [OR] (95% CI)
Age		
18–39 years	Reference	
40–59 years	1.12 (0.68, 2.01)	2.32 (0.92, 4.02)
60+ years	1.08 (0.50, 2.33)	0.71 (0.21, 2.41)
Gender		
Female	Reference	
Male	0.42 (0.1, 2.31)	1.33 (0.79, 3.62)
Identify as LGBTQ+		
No	Reference	
Yes	0.83 (0.13, 5.17)	3.00 (0.36, 12.92)
Race/Ethnicity		
White	Reference	
Black	0.52 (0.21, 1.72)	1.22 (1.08, 1.76)*
Hispanic/Latino	1.40 (0.14, 3.11)	2.04 (1.12, 6.51)*
Others	0.53 (0.14, 2.43)	2.03 (1.31, 10.23)*
Citizenship status		
US citizen	Reference	
Lawfully present/non-immigrant	0.62 (0.30, 1.27)	0.18 (0.05, 0.63)*
Undocumented	0.82 (0.23, 1.51)	3.43 (1.98, 9.53)*
Primary language		
English	Reference	
Spanish	3.12 (1.31, 6.40)*	5.16 (1.85, 9.10)*

* Represents statistically significant values based on Type 1 error rate set at 5%, ie, P-values less than 0.05.

*OR*, odds ratio; *CI*, confidence interval; *LGBTQ+*, lesbian gay, bisexual, transgender, queer and other; *US*, United States.
